# Huh, what did they say again? The influence of task interruption position and workload on auditory-verbal memory performance

**DOI:** 10.1186/s41235-026-00709-x

**Published:** 2026-02-16

**Authors:** Sandra Hensen, Iring Koch, Abbie Jin, Patricia Hirsch

**Affiliations:** Institute of Psychology, RWTH Aachen University, Jaegerstrasse 17/19, 52066 Aachen, Germany

**Keywords:** Interruption, Memory, Workload

## Abstract

The harmful effects of task interruptions on performance in discrete visual-manual reaction-time (RT) tasks are well investigated, but the impact on continuous auditory-verbal memory tasks has received comparably less attention. In the present study, the encoding phase of an auditory-verbal free recall task was interrupted by visual–manual RT tasks. To examine which factors influence the disruptiveness of an interruption during the encoding phase, we manipulated the cognitive workload (low vs. high) associated with the interruption task and the position (early vs. late) of the intervening interruption tasks. Moreover, we manipulated the central code (verbal vs. spatial) in the high-workload interruption task. Results showed decreased free recall with late and high-workload interruptions compared to early and low-workload interruptions. However, the variation of central codes did not influence free recall in the high-workload interruption task. We also examined task trade-offs and found worse performance in the interruption task itself compared to performing it as a single task. These results suggest that memory maintenance in the memory task interferes with response selection in the interruption task. Further, we assume that early interruptions of memory encoding are less harmful than later ones because less primary task information needs to be maintained during interruption task processing. Overall, our study revealed that task interruptions lead to information loss in auditory-verbal memory encoding, highlighting the importance of minimizing interruption costs in work environments where effective communication and information exchange are crucial.

## Significance Statement

In our modern everyday life, task interruptions occur frequently. Especially in work environments such as healthcare, where medical staff must manage competing demands in time-critical situations while maintaining effective verbal communication and patient-related information, interruptions can lead to information loss and impaired patient safety. Despite extensive research on how interruptions affect discrete visual-manual reaction-time (RT) tasks, their impact on auditory-verbal memory, which is critical for verbal communication and information exchange, has been neglected. Our research addresses this gap by investigating how different types of interruptions influence memory performance in an auditory-verbal free recall task. Specifically, we investigated if the cognitive workload of the interruption task and the position of an interruption (early or late phase of an information exchange) influence the size of the interruption costs. Moreover, we examined if the similarity of the primary and interruption task impacts memory performance. We found that late and high-workload interruptions significantly disrupt memory performance compared to early and low-workload interruptions, emphasizing the vulnerability of auditory-verbal memory to interference. Furthermore, results showed that interruptions not only impair recall but also decrease performance in the interrupting task itself. These findings have direct implications for real-world settings where verbal information must be remembered under conditions of frequent, unavoidable interruptions, such as in polytrauma care. By examining the cognitive mechanisms underlying these interruption costs, our study highlights the importance of designing strategies to avoid interruption or minimizing the negative impact of interruptions on memory, improving information exchange and decision-making in critical work environments.

## Introduction

Interruptions of ongoing tasks are common in our technology-driven everyday life, including both personal and work environments. All of us experienced situations where a task is abruptly halted by another demand, resulting in its postponement and later resumption. For example, in healthcare settings medical staff might find themselves interrupted by urgent calls or alerts while completing a task. In the context of patient handovers in polytrauma care, interruptions during these critical auditory-verbal information exchanges can potentially lead to communication errors and information loss, resulting in compromised patient safety (see e.g., Grundgeiger & Sanderson, 2009; Sanderson et al., 2019, for reviews). The present study aimed to investigate the cognitive mechanisms of these possibly harmful effects of task interruptions on memory performance with auditory-verbal information. To this end, we examined the impact of both early vs. late and high- vs. low-workload interruptions on recall performance in a primary free recall memory task. Additionally, we explored whether the recall probability of information was affected by an overlap of the central codes (verbal vs. spatial) used in the primary memory task and the high-workload intervening interruption task. Lastly, to examine potential trade-offs in task performance, we investigated whether performance in the interruption task itself was negatively impacted by the interruption context (i.e., memory maintenance in the ongoing primary memory task).

### Investigating memory effects with free recall memory tasks

A free recall memory task typically consists of an encoding phase, a distractor task, and a retrieval phase and is a common task for investigating the impact of simultaneous multitasking (i.e., dual-task effects) on memory (see e.g., Baddeley et al., 1984; Craik et al., 1996; Fernandes & Moscovitch, 2000; Hensen et al., 2024; Naveh-Benjamin et al., 2000). In dual-task situations, a primary task (e.g., encoding words) and a secondary task (e.g., a reaction-time [RT] task) are performed simultaneously. Performance in dual-task situations is compared to performance in single-task situations (i.e., isolated task performance) to assess dual-task costs. The dual-task situation can be implemented either in the encoding phase or the retrieval phase. During the encoding phase, visual or auditory word lists are encoded and need to be maintained. The encoding phase is followed by a distractor activity (e.g., counting backward by threes from a randomly chosen number) to eliminate any recency effects (i.e., participants often recall the last remembered items more accurately; see e.g., Delaney et al., 2010). After the distractor activity, participants recall the to-be-remembered words in any order as quickly as possible during the retrieval phase (i.e., a delayed memory test).

In the present study, we used such a free recall memory task to investigate memory effects, but instead of simultaneous multitasking (i.e., dual tasks) we implemented sequential multitasking. Sequential multitasking can be implemented either by switching repeatedly between the stimulus–response mappings of two fully completed tasks (i.e., task switching) or by interrupting an ongoing primary task, performing an intervening interruption task and resuming the unfinished primary task after interruption task completion (i.e., task interruption^1^; see Hirsch et al., 2022, for an overview of the time course of a task switch and a task interruption). In the present study, the encoding phase of an ongoing primary memory task was temporarily paused in order to perform an interruption task. After completion of the interruption task, the encoding phase of the ongoing primary memory task was resumed and completed, followed by a distractor activity and retrieval phase. As the previously encoded memory items need to be maintained during interruption task processing and the encoding phase of memory task is not completed, methodologically the cognitive demands of this experimental setup align more closely to that of a task interruption than a task switch. However, please note that our paradigm differs from standard task interruption studies, which often involve two discrete tasks (i.e., tasks that require manual responses after each stimulus) with specific task-related goals to remember (i.e., prospective memory tasks; see e.g., Altmann & Trafton, 2007). Thus, the novel aspect of the present study is that it combined a continuous free recall memory task (without specific task-related goals; an episodic memory task) with a discrete RT task in an interruption paradigm.^2^

### Detrimental effect of task interruptions on the ongoing primary task

Previous research showed that interruptions lead to a general performance decline in the ongoing primary task (see e.g., Edwards & Gronlund, 1998; Gillie & Broadbent, 1989; Hirsch et al., 2024, for examples with discrete tasks; see also Varao-Sousa et al., 2018, for an example with a continuous audio book questionnaire primary task). One general explanation for the harmful effects of task interruptions on primary task performance refers to the need to shift the focus of attention to the intervening interruption task, leading to a temporal disengagement from all task-related information from the primary task (e.g., task-related goals, see Altmann & Trafton, 2007; or memory items, see Bae & Luck, 2018; Zickerick et al., 2021). When resuming the primary task, the redirection of attention and the reactivation of the task-related information cause performance costs, resulting in an overall decrease in both speed and accuracy, especially in more complex tasks (see e.g., Couffe & Michael, 2017; Trafton & Monk, 2007, for reviews). These costs can be reduced through working memory (WM)^3^ maintenance mechanisms, like e.g., articulatory rehearsal for verbal material (e.g., Baddeley, 1986) or general attentional refreshing (e.g., Cowan, 1999). Both mechanisms ensure that task-related information of the ongoing primary task is kept in an active state to be maintained, but depending on the interruption task, using WM maintenance mechanisms might not be possible during the interruption phase (see e.g., Couffe & Michael, 2017).

In the context of a free recall memory task with an interruption during the encoding phase and a delayed memory test, it is important to mention that WM maintenance is suggested to lay the ground for episodic long-term memory (LTM) formation and can improve performance in LTM tests (e.g., Hartshorne & Makovski, 2019, for a meta-analysis; see also Bartsch et al., 2024, for a review about the relationship between WM and LTM). For example, Bartsch et al. (2018) investigated whether the WM maintenance mechanisms of refreshing and elaboration (i.e., the process of encoding information more deeply and linking it to the existing network of associations) improve WM and LTM performance. In their study, participants memorized nouns in a serial order, which were either refreshed or elaborated in different conditions. An immediate memory test was performed and after a distractor task, a delayed memory test took place. Results showed that delayed LTM performance was better when the memory items were elaborated, compared to conditions without elaboration, suggesting that LTM can benefit from WM maintenance. Thus, in a free recall memory task with a delayed memory test, an interruption during encoding might impair episodic memory performance because of interference with WM maintenance, due to limited attentional resources.

Overall, previous empirical research revealed factors that influence the disruptiveness of task interruptions in different tasks and with different measures (e.g., task completion time, accuracy, situation awareness, etc.). Such factors are, for example, the complexity of both the primary and interruption tasks (e.g., Speier et al., 1999) and the temporal interruption position (e.g., Bailey & Konstant, 2006). Also, central code overlap between both tasks (e.g., spatial vs. verbal processing; Wickens, 2008) was suggested as an influencing factor for interruption costs (e.g., Lee et al., 2018; Zhou et al., 2024).

### Task interruptions and task complexity

Studies that investigated the effect of primary task complexity showed that interruptions are more harmful for complex primary tasks (e.g., in terms of a more complex decision component) compared to simple, repetitive primary tasks (see e.g., Speier et al., 1999). Similar results were found with increasing interruption task complexity (see e.g., Radovic & Manzey, 2022; Zickerick et al., 2021).

For example, Zickerick et al. (2021) asked participants to keep the orientation of two laterally presented bars in WM, while they were interrupted by either a low- or high-workload arithmetic task. They found that primary task WM performance was worse with the high-workload interruption compared to the low-workload interruption. The authors suggested that during the interruption phase, participants already engaged in rehearsing the WM item to resume the ongoing primary task. Thus, processing of both tasks overlaps in time, resulting in a compromised attentional refocusing on the WM items, especially with the more demanding high-workload arithmetic task. In the present study, we aimed to examine the effect of interruption task complexity with an auditory-verbal LTM task, by interrupting the task with either a low-workload interruption task (i.e., no decision component) or a high-workload interruption task.

### Task interruptions and overlapping central codes

Another potentially influencing factor for the disruptiveness of interruptions on primary task performance refers to whether the ongoing primary task and the intervening interruption task share central codes. According to multiple resource theory (Wickens, 2008), the central code refers to whether tasks require spatial or verbal processing. Resource theories postulate that two tasks interfere with each other to the degree they need to share resources (see Koch et al., 2018, for a general review of multitasking). Hence, two tasks that both require verbal processing should interfere more with each other than two tasks that rely on verbal and spatial processing.

However, previous task interruption studies investigating the effect of code overlap showed mixed results (e.g., Lee et al., 2018; Radovic & Manzey, 2022; Zhou et al., 2024). For example, Zhou et al. (2024) showed that situation awareness of traffic controllers was worse when the ongoing primary tasks (visual air traffic control task or auditory air-ground communication task) and the interruption tasks (visual image identification task or auditory telephone-answering task) shared the same modalities, compared to different modalities in both tasks. These findings imply that interference is increased when two tasks have stronger resource overlap. In contrast, Radovic and Manzey (2022; Experiment 2) could not find a significant impact of central code overlap on primary task resumption with a verbal procedural ongoing primary task and either a verbal or spatial classification task as intervening interruption task. As the effect of central code overlap has not yet been investigated with an auditory-verbal free recall memory task as ongoing primary task, we implemented a visual-manual interruption task requiring either verbal or spatial central codes in our study. The aim was to re-assess the role of central code overlap on auditory LTM performance in a task interruption paradigm.

### Task interruptions and the temporal interruption position

Besides specific characteristics of the primary task and interruption task, also the temporal interruption position is an important factor for the disruptiveness of task interruptions. To examine interruption position effects, several studies used procedural tasks consisting of a predefined sequence of subtasks (e.g., Hirsch et al., 2025; Radovic & Manzey, 2019). In these procedural tasks, interruptions were less harmful when a task was interrupted between subtasks compared to within a subtask (e.g., Bailey & Konstant, 2006).

Theoretically, interruptions in the context of episodic memory tasks should be less harmful at the beginning of a primary task compared to the middle or end because maintenance for less memory items needs to take place during interruption task processing. However, such differences in episodic memory performance between early, middle, and late interruptions were not investigated so far. In our study, we aimed to fill this gap by examining the influence of the temporal interruption position in the context of auditory-verbal free recall.

### Detrimental effect of task interruptions in the intervening interruption task

Previous research mainly focused on primary task performance only, but in our opinion, it is also important to consider performance in the interruption task itself. Coming back to the healthcare setting, if an exchange of patient-related information (ongoing primary task) is interrupted by an emergency (e.g., the patient is choking and needs immediate assistance; intervening interruption task), it is not only important to remember information of the ongoing primary task, but also performance in the interruption task is crucial for patient safety. Only a few studies analyzed interruption task performance in the context of task interruptions, with mixed results (see e.g., Brazzolotto et al., 2022; Hirsch et al., 2024; Radovic & Manzey, 2022).

For example, in the study mentioned earlier, Radovic and Manzey (2022) examined interruption costs between a procedural primary task (including verbal rehearsal) and different interruption tasks (verbal or spatial WM task in Experiment 1 and verbal or spatial classification task in Experiment 2). Performance of the interruption task was compared in interruption conditions (i.e., the task interrupts the ongoing primary task) with single-task conditions (i.e., the same task is performed without an interruption context). Results showed no significant impact of the interruption context on performance in the WM task, but performance of the classification task was overall worse with the interruption context compared to the single-task context. This indicates that not only primary task performance can be negatively influenced by the interruption context, but there might be trade-offs in the interruption task itself. In summary, investigating the performance of the intervening interruption task itself is important to gain a better understanding of the interrelation between primary task and interruption task.

### Present study

The aim of the present study was to investigate the effects of early and late task interruptions as well as high- and low-workload task interruptions on auditory-verbal memory performance. Moreover, we examined the influence of central code overlap (verbal vs. spatial) between the primary task and a high-workload interruption task. We used an auditory-verbal free recall memory task with a delayed memory test as an ongoing primary task. As intervening interruption tasks, we employed two different visual-manual RT tasks (high workload and low workload) that interrupted the encoding phase of the primary memory task either early or late.

For the high-workload interruption task, we employed a cognitively demanding visual-manual four-choice spatial Stroop task. This task requires the categorization of the spatial meaning of targets (e.g., the words “left” vs. “right”) that appear at different spatial positions on the screen (e.g., on the left or right of screen center). Spatial incongruence of target meaning (e.g., “left”) and irrelevant spatial position (e.g., right) typically causes performance costs relative to spatial congruence (e.g., “left” on left position; congruency effect^4^;see Lu & Proctor, 1995, for a review). While processing the spatial Stroop task as interruption task, memory maintenance processes should be limited due to interference. To examine whether verbal stimuli interfere more with verbal memory relative to spatial stimuli, we manipulated the central code overlap (Wickens, 2008) between the ongoing primary task and high-workload interruption task by either using spatial words (verbal) or arrows (spatial) as target stimuli.

The low-workload interruption task was a simple, repetitive (i.e., no decision component) alternating tapping task that was not supposed to create strong interruption costs in the primary memory task compared to the high-workload interruption task. We decided to implement a low-workload task instead of just a pause with silence to keep participants engaged in the experiment.

Lastly, we analyzed performance in the high-workload interruption task itself (i.e., the spatial Stroop task) to assess whether performing the task within an interruption context (i.e., during maintenance of memory items) impaired task performance. To this end, we compared spatial Stroop performance in the interruption conditions with performance in a single-task condition of the same task. Apart from expecting the standard congruency effect as a manipulation check, we did not have strong predictions for this task.

For the primary memory task, we expected worse recall performance in the primary memory task when the encoding phase is interrupted by a high-workload interruption task compared to a low-workload interruption task. Further, we hypothesized that a late interruption leads to a larger memory performance decline compared to an early interruption. Regarding overlapping central codes, we expected increased interference between both tasks with words (verbal) compared to arrows (spatial) used as stimulus material in the high-workload interruption task, resulting in worse recall performance.

## Method

### Stimuli, tasks, and responses

The ongoing primary task was an auditory-verbal free recall memory task, in which participants encoded auditory word lists and vocally recalled the to-be-remembered words in a later retrieval phase after performing a distractor activity (counting backwards by threes) to eliminate any recency effects. The high-workload intervening interruption task was a visual-manual spatial Stroop task, in which participants classified the spatial meaning of a target (word or arrow) while ignoring the spatial position of the target on the screen. The low-workload interruption task was an alternating tapping task, in which participants were asked to manually respond to the location of a visually presented square that alternated from the left to the right side on the screen.

For the primary task, 190 German two- and three-syllable words were used as auditory stimulus material. To enhance the applicability of our findings into real-world settings where individuals primarily engage with domain-specific information (e.g., medical settings), we used auditory stimuli relevant to clinical psychology, such as names, symptoms of mental disorders, and psychological concepts. The words were primarily selected from the Diagnostic and Statistical Manual of Mental Disorders (American Psychiatric Association, 2013; e.g., “Depression,” “Emotion,” “Memory,” etc.). A text-to-speech converter with a female voice was used to generate the audio files. The duration of each audio file was edited to be one second. Nine lists with 15 words and two lists with five words (for practice, see below) were created by randomly assigning each word to a list, without mixing the two- and three-syllable words. For each participant, the word order in all lists was randomized. A 600 Hz “beep” sound signaled the start of the retrieval phase of the primary memory task.

For the visual interruption tasks, we used a fixation cross (+), randomly generated three-digit numbers, either the German location words “LINKS,” “RECHTS,” “OBEN,” and “UNTEN” (left, right, up, and down) or arrows pointing left, right, up and down, and a square as visual stimulus material. As error feedback, the German word “FEHLER” (error) was visually displayed. The visual stimuli were presented in black on a white background in “Arial” font. The height of the fixation cross, numbers, and error feedback was 2 cm; the height of the location words was 3 cm, the size of the arrows 3 × 6 cm, and the size of the square 6 × 6 cm. The fixation cross, numbers, and error feedback were positioned in the center of the screen. The position of the location words “LINKS” and “RECHTS” or the left- and right-pointing arrows changed horizontally (50%/50%), with 6 cm to the left or right side of the screen center (e.g., the word “LINKS” or the left-pointing arrow was displayed half the time on the left side and half the time on the right side of the screen). The position of the words “OBEN” and “UNTEN” or the up- and down-pointing arrows changed vertically (50%/50%), positioned 6 cm above or below the screen center (e.g., the word “OBEN” or the up-pointing arrow was displayed half the time in the upper part and half the time in the lower part of the screen). The position of the square also changed horizontally (50%/50%), with 6 cm to either the left or right side of the screen center. All the sizes were measured on a 27-inch screen.

Vocal responses were recorded via microphone. Manual responses were performed via keyboard, using the keys “Y” for the response left, “X” for the response right, arrow key “up” for the response up, and “down” for the response down with the middle and index fingers of the left and right hand. The experiment was programmed and run using PsychoPy 3 (Peirce et al., 2019), version 2021.1.4 on a windows 10 computer.

### Procedure

Instructions were given in verbal and written form, emphasizing speed and accuracy in all tasks. Before the main experiment started, participants filled out an informed consent and data protection form as well as a demographic questionnaire.

**Fig. 1:**
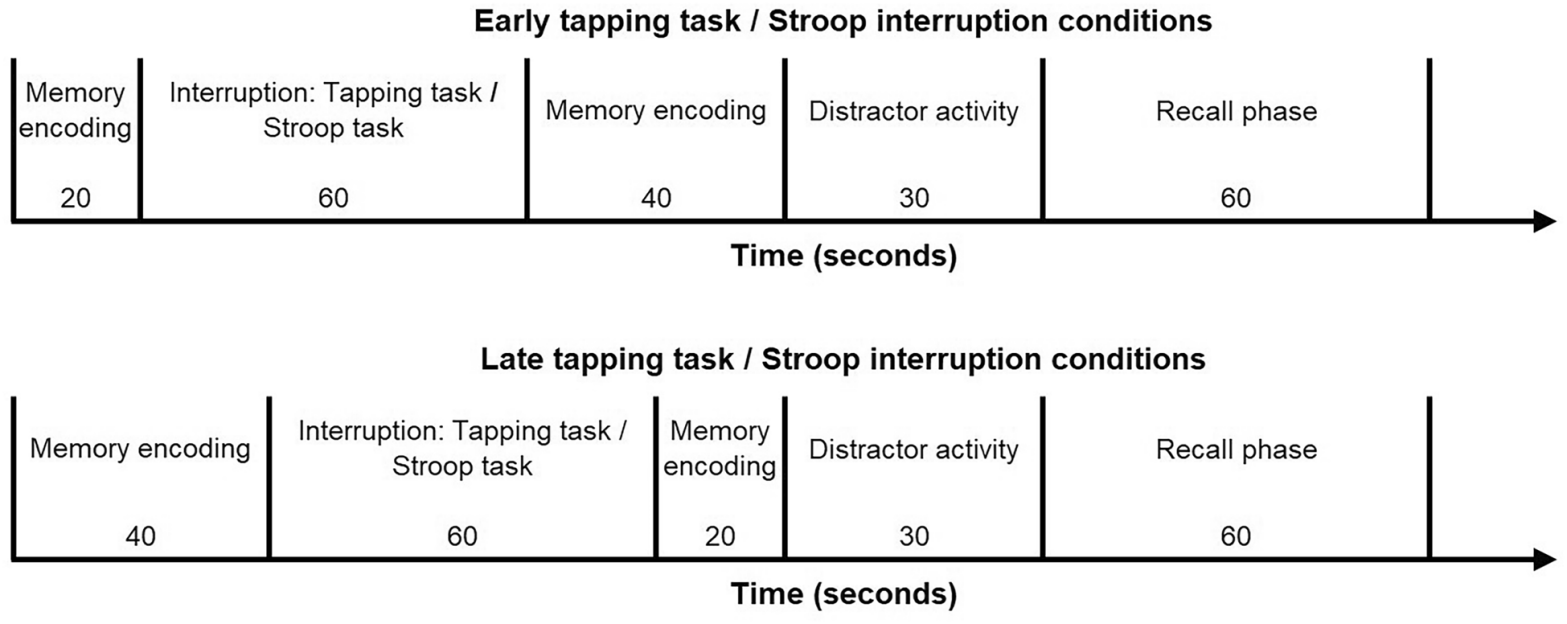
Schematic illustration of the procedure in the different experimental conditions (early tapping task and early spatial Stroop interruption conditions, as well as late tapping task and late spatial Stroop interruption conditions; excluding the spatial Stroop single-task condition). *Note.* While encoding, the memory items were presented at a 4-s rate (i.e., 5 words in 20 s or 10 words in 40 s). In the 60-s interruption phase, memory encoding was interrupted by the tapping task or spatial Stroop task (with words or arrows). Participants performed 30 tapping task trials or 30 spatial Stroop trials, as the duration of one trial was 2 s in both tasks. During the distractor activity, participants counted backwards by threes for 30 s. In the recall phase, participants had 60 s to recall the previously encoded memory items

The main experiment included a single-task condition for the spatial Stroop task, in which the task was performed alone (i.e., without interruption context). Further, there were two different interruption conditions, in which the encoding phase of the memory task was interrupted either early or late by the low-workload alternating tapping task (early vs. late tapping task interruption condition) or the high-workload spatial Stroop task (early vs. late spatial Stroop interruption condition; see Fig. 1 for an overview of the conditions). Participants were randomly assigned to one of two different groups with different stimulus material used in the spatial Stroop task (word group [verbal] vs. arrow group [spatial]; i.e., between-subjects variation).

In the primary memory task, 15 words were encoded with one word serially presented every 4 s, while a fixation cross remained on the screen. The primary memory task was interrupted after either five words (early interruption) or 10 words (late interruption) were encoded. The interruption task was either the low-workload alternating tapping task (tapping task interruption condition) or the high-workload spatial Stroop task (spatial Stroop interruption condition). After completing 30 trials of the interruption task (60 s interruption), the encoding phase was resumed, and the remaining 10 words (early interruption) or five words (late interruption) were presented. Then, participants engaged in a 30-s distractor activity. Afterward, a tone signaled the start of a 60-s recall period, in which participants verbally recalled the list of 15 words in any order.

For the low-workload alternating tapping task, each trial started with the presentation of a fixation cross and a square, which was either on the left or right side of the screen. The square remained on the screen for 1500 ms. After this time, the square disappeared, and the fixation cross persisted for 500 ms. The square then switched to the opposite location for the next trial. In case of an error or timeout (no response within 1500 ms), participants received visual error feedback for 300 ms, followed by the fixation cross for 200 ms. In total, the length of one trial was 2000 ms and participants completed 30 trials per block.

For the spatial Stroop task, each trial started with the presentation of a fixation cross and a location stimulus (word or arrow) that remained on the screen for 1500 ms. After 1500 ms, the location stimulus disappeared, and the fixation cross persisted for 500 ms until the start of the next trial. In case of an error or timeout (no response within 1500 ms), participants received visual error feedback for 300 ms, followed by the fixation cross for 200 ms. Thus, the length of one trial was 2000 ms, like in the alternating tapping task. Stimuli were presented randomly, ensuring equal frequency, with each participant completing 30 trials per block, comprising 15 congruent and 15 incongruent trials.

Three practice blocks were accomplished to familiarize participants with the experimental tasks. Participants performed a practice block of the spatial Stroop single-task condition first (16 trials), followed by two practice blocks of the memory task with five-element word lists that were either interrupted by 16 trials of the alternating tapping task (tapping task interruption condition) or spatial Stroop task (spatial Stroop interruption condition), after three words were encoded. After completing the high- or low-workload interruption tasks, the remaining two words were encoded, followed by a 15-s distractor activity and 20-s retrieval phase. Each participant completed three experimental blocks for each condition (early and late tapping task interruption condition, spatial Stroop single-task condition, early and late spatial Stroop interruption condition), totaling 15 experimental blocks. The order of these blocks was counterbalanced across participants in both groups (word vs. arrow), using a Latin square design. Furthermore, it was counterbalanced across participants in both groups which of the wordlists were used in either the early vs. late tapping task interruption conditions or early vs. late spatial Stroop interruption conditions. The experiment took approx. 60 min.

### Design

For the ongoing primary memory task, we specified a 2 × 2 × 2 mixed factorial design. The independent within-subject variables were interruption task complexity (low-workload tapping task vs. high-workload spatial Stroop task) and interruption position (early vs. late). The between-subject variable was central code (word [verbal] vs. arrow [spatial] group in the high-workload spatial Stroop task). The dependent variable was mean recall total (a percentage of correctly recalled words).

For the spatial Stroop task, that is, the high-workload interruption task, we specified a 2 × 2 × 2 mixed factorial design with the independent within-subject variables interruption context (single-task vs. interruption of the memory task), congruency (congruent vs. incongruent trials), and the between-subject variable central code (word [verbal] vs. arrow [spatial] group). The dependent variables were RT and error rates.

### Participants

Seventy-two recruited psychology students (44 females; mean age = 23.96 years, *SD* = 5.01) took part in the experiment. All participants were native German speakers and reported normal or corrected-to-normal vision and hearing acuity. All gave their informed consent for participation and received partial course credit or monetary compensation (10€ per hour).

To determine the sample size, we conducted a power analysis using MorePower 6.0 (Campbell & Thompson, 2012). Power was calculated for an analysis of variance (ANOVA) with the most complex design: two within-subject factors (all with two levels) and one between-subject factor (two levels). As effects of interest, all three main effects were selected (main effects of interruption task complexity, interruption position, and central code) based on our most important hypotheses. In the power analysis, we did not focus on any interaction effects, as we had no strong predictions for these effects. We specified a medium to large effect size (ηp^2^ =.10). With an alpha of 0.05, the power analysis showed that at least 36 participants per group were required for a power of 0.8.

## Results

The data were analyzed using IBM SPSS, and all analyses were calculated at α =.05. Practice blocks were discarded from all analyses. We report the results separately for the primary memory task and the interruption spatial Stroop task. Data from the alternating tapping task were not analyzed. Participants’ datasets were excluded from all analyses when they recalled zero words in one or more block(s) of the memory task, or their error rate was above 40% in one or more block(s) of the spatial Stroop task (n = 2). For the RT analysis of the spatial Stroop task, trials with an erroneous response (4.4%) and trials deviating more than ± 3 standard deviations from each participant’s individual mean RT per condition (0.9%) were additionally eliminated from the remaining data.

### Primary memory task

**Fig. 2:**
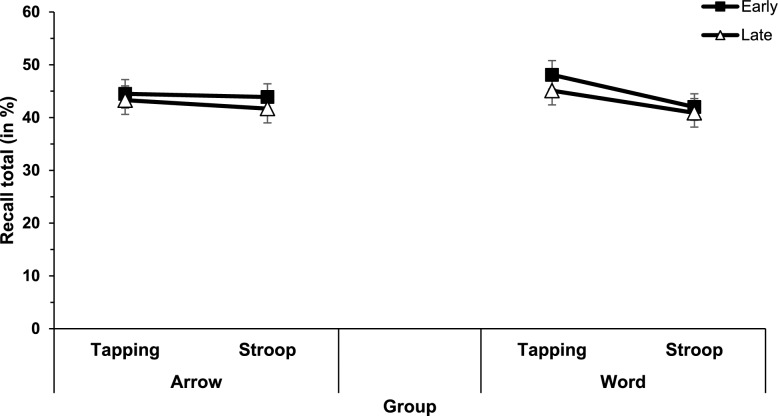
Mean recall total (in %) of the memory task for the different interruption conditions (interruption task complexity and interruption position) separated by central code (Stroop task with words [verbal] vs. arrows [spatial]). Error bars show standard errors

A 2 × 2 × 2 mixed ANOVA with the independent variables interruption task complexity (low-workload tapping task vs. high-workload spatial Stroop task), interruption position (early vs. late), and central code (word [verbal] vs. arrow [spatial] group) was conducted for recall total (see Fig. 2). Results showed a significant main effect of interruption task complexity (*F*(1,70) = 8.42, *p* =.005, η_p_^2^ =.108), indicating reduced recall total when being interrupted by the high-workload Stroop task compared to the low-workload tapping task (42.1% vs. 45.3% or 6.3 words vs. 6.8 words). Also, the main effect of interruption position was significant (*F*(1,70) = 5.02, *p* =.028, η_p_^2^ =.067), with reduced recall total for the late interruption condition compared to the early interruption condition (42.8% vs. 45.6% or 6.4 words vs. 6.8 words). The main effect of central code was not significant (*F* < 1; word group 44.0% or 6.6 words; arrow group 43.4% or 6.5 words).

All interactions were not significant. There was a numerical trend for the interaction between interruption task complexity and central code (*F*(1,70) = 3.55, *p* =.064; word group 5.1% or 0.8 words interruption task complexity costs; arrow group 1.1% or 0.2 words interruption task complexity costs), but given the non-significance of the interaction, we refrain from interpreting this numerical trend. All other interactions, *F* < 1.

We also analyzed recall probabilities of memory items depending on their serial position in the list (beginning, middle, and end) on an exploratory basis to ensure that differences in the different interruption conditions are not due to serial position effects in just one of the conditions. We found overall better recall probabilities for items at the beginning of the list (52.9% or 7.9 words) compared to middle (38.9% or 5.8 words) or end of list items (39.8% or 5.8 words) reflecting a primacy effect, but this effect was not modulated by the different interruption conditions (*p* =.364). Detailed results can be found in appendix.

### Intervening interruption task (spatial stroop task)

**Fig. 3:**
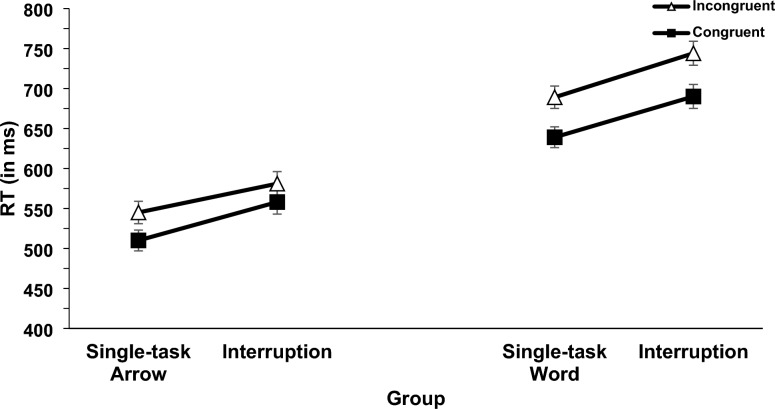
Mean RT (in ms) of the spatial Stroop task. Error bars show standard errors

**Fig. 4:**
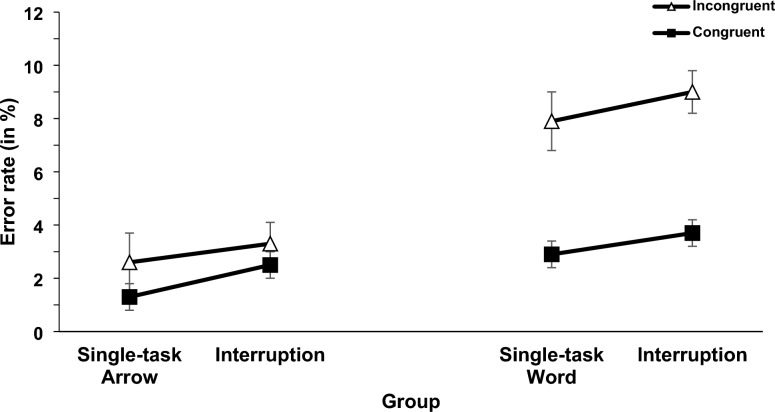
Mean error rates (in %) of the spatial Stroop task. Error bars show standard errors

Two 2 × 2 × 2 mixed ANOVAs with the independent variables interruption context (single-task vs. interruption of the memory task), congruency (congruent vs. incongruent), and central code (word [verbal] vs. arrow [spatial] group) were conducted for RT and error rates (see Fig. 3 and 4)^5^ For RT there was a significant main effect of interruption (*F*(1,70) = 59.12, *p* <.001, η_p_^2^ =.458). RT was longer in the interruption condition compared to the single-task condition (645 ms vs. 590 ms). This effect was not significant for error rates (*F*(1,70) = 3.85, *p* =.054) but showed the same pattern (interruption 4.7% errors; single-task 3.8% errors). Further, the main effect of congruency was significant for RT (*F*(1,70) = 187.33, *p* <.001, η_p_^2^ =.728) and error rates (*F*(1,70) = 43.37, *p* <.001, η_p_^2^ =.383), indicating longer RT and increased error rates in incongruent than in congruent trials (639 ms vs. 596 ms; 5.8% vs. 2.6%). Also, the main effect of central code was significant for RT (*F*(1,70) = 73.60, *p* <.001, η_p_^2^ =.513) and error rates (*F*(1,70) = 37.76, *p* <.001, η_p_^2^ =.350). RT was longer and error rates were increased in the word group compared to the arrow group (688 ms vs. 547 ms; 6.1% vs. 2.4%).

Moreover, the interaction between congruency and central code was significant for RT (*F*(1,70) = 16.24, *p* <.001, η_p_^2^ =.188) and error rates (*F*(1,70) = 17.01, *p* <.001, η_p_^2^ =.195), indicating an increased congruency effect in the word group compared to the arrow group (55 ms vs. 30 ms; 5.2% vs. 1.2%). All other interactions were not significant (interaction between interruption and central code for RT, *F*(1,70) = 2.37, *p* =.128; three-way interaction for RT, (*F*(1,70) = 3.83, *p* =.067, all other interactions for RT and ER, *F* < 1).

## General discussion

The present study investigated the impact of task interruptions on free recall performance in a delayed memory test. We examined the impact of both early and late as well as high- and low-workload interruptions with visual-manual RT tasks on recall performance in an auditory-verbal free recall task. Furthermore, we analyzed the effect of central code overlap (spatial vs. verbal) between the primary memory task and the high-workload interruption task on memory performance.

Results showed that high-workload interruptions were more harmful for recall performance compared to low-workload interruptions. Further, in both interruption task conditions, late interruptions led to a significant performance decline in recall accuracy relative to early interruptions. Central code overlap between tasks had no significant impact on memory performance.

We also analyzed performance of the high-workload spatial Stroop task, as we were interested in whether performance in the interruption task itself was negatively impacted by the interruption context (i.e., memory maintenance in the primary memory task). Besides the standard congruency effect, results showed that performance in the high-workload interruption task was impaired in the interruption condition relative to performing the task in a single-task condition.

### What makes interruptions disruptive?

First, the serial position analysis showed a primacy effect in both the low-workload tapping task and high-workload spatial Stroop interruption conditions, indicating that the observed decreased recall performance in the primary memory task with high-workload interruptions compared to low-workload interruptions was not due to serial position effects in just one of the conditions. Together with the finding that performance in the high-workload spatial Stroop task itself was worse when it was performed in an interruption context compared to performing it in a single-task context (independent from the used stimulus material), we assume that memory maintenance in the primary task interferes with response selection in the interruption task. More specifically, we suggest that memory maintenance processes (i.e., rehearsal, refreshing, or elaboration) either interfere with retrieving the task-set rules^6^ that are needed for response selection in the interruption task, or with repeated Stroop conflict resolution due to conflicts at target identification and response selection in incongruent trials (Botvinick et al., 2001). Because it was shown in previous studies that WM maintenance lays ground for LTM performance (e.g., Bartsch et al., 2018; Hartshorne & Makovski, 2019, for a meta-analysis; see also Bartsch et al., 2024, for a review), we believe that the performance decline that we observed in our LTM test is due to interference between memory maintenance and response selection processes.

This mutual interference between memory maintenance and response selection processes would explain why performance suffers in both the primary memory task and spatial Stroop interruption task, and why performance in the memory task was better when interrupted by the low-workload tapping task compared to the high-workload spatial Stroop task. In the alternating tapping task, participants only needed to remember whether they pressed the left or right key last and alternate to the opposite key. Thus, this task does not include a strong decision component. In contrast, with the spatial Stroop task as interruption task, processes in both tasks compete and interfere more with each other due to the more complex response selection processes and Stroop conflict resolution (see also e.g., Dodhia & Dismuke, 2009; Einstein et al., 2003; for studies that suggested interference between maintenance processes in prospective memory tasks and response selection processes in interruption tasks). As most previous studies used discrete visual-manual tasks as primary tasks in task interruption research, this interruption effect in an auditory-verbal free recall task is a novel contribution of the present study.

Further, the finding that recall performance in the primary memory task was worse in the late interruption condition compared to the early interruption condition is in line with the idea of interference between memory maintenance in the primary task and response selection in the interruption task. When less primary task information (e.g., five memory items vs. 10 memory items in case of our study) needs to be maintained during interruption task processing, there are less opportunities for interference between both tasks. So far, there were no studies that investigated the impact of interruption position on episodic LTM (as most studies used procedural primary memory tasks; e.g., Bailey & Konstant, 2006); thus, this finding is a novel contribution to interruption research.

Note that we also examined the influence of central code overlap between primary memory task and interruption task performance, but we could not find a significant difference in recall performance with the verbal and spatial stimulus material used in the spatial Stroop interruption task. We expected more interference when the primary memory task and spatial Stroop interruption task both involve verbal processing, but as in the study of Radovic and Manzey (2022), there was no clear evidence for such a detrimental effect of central code overlap. Radovic and Manzey (2022) argued that their verbal primary task might depend more on general memory processes, which are largely independent of specific processing codes or specific memory subsystems. This could also be the case for our primary task. Another explanation could be that even with the verbal stimulus material (word group), the goal of the spatial Stroop task was still to classify the spatial meaning of a location word. Thus, also with the verbal stimulus material, there was still a strong spatial component in the task. Overall, the only significant material effect we found was generally worse spatial Stroop task performance in the group with verbal stimulus material compared to spatial stimulus material, independent from the interruption context. This result suggests that verbal material is simply more difficult to process than spatial stimulus material, as we found a similar pattern in a previous dual-task study with the same tasks and verbal vs. spatial groups (Hensen et al., 2024,7).

### Task interruptions and consequences for applied scenarios

Coming back to the healthcare scenario described in the introduction, what conclusions can we draw from our findings? First, our study showed that high-workload interruptions (i.e., any task that involves a strong decision component) should be prevented by medical staff, as they have a significantly greater impact on auditory-verbal LTM performance compared to low-workload interruptions. Note that even if the absolute difference between the low-workload and high-workload interruption conditions in our study was numerically small (0.5 words), our analysis still showed a medium effect size. In addition, information remembered in real-life scenarios is typically more complex and meaningful, so that even small reductions in memory performance could have substantial practical implications. Secondly, our results indicate that interruptions at a later stage of an auditory-verbal information exchange are especially critical for memory performance and should be avoided to prevent information loss of crucial patient-related information.

Moreover, our results suggest that not only information loss could be critical in the context of healthcare, but also impaired performance in the intervening interruption task itself (e.g., a care task) due to memory maintenance in the primary task. Note that the spatial Stroop task used in the present study is a well-established task often used in cognitive psychology to examine cognitive control processes. At the same time, it is important to note that the Stroop task might not fully capture the nature of real-life interruptions, especially in high-stake environments like healthcare, where task interruptions could also involve emotional components or induce high-stress situations due to the severity of injuries.

As the nature of task interruptions is that they are sometimes not avoidable, support systems like an automatic speech recognition system that encodes spoken words and shows the most important information on a screen are desirable. With such support systems, not only information loss could be prevented due to automatic documentation, but also performance of care tasks and decision-making can be facilitated as memory load can be reduced for medical staff due to an external opportunity to store patient-related information.

### Limitations and future research

One limitation of our study is that we cannot distinguish between the different maintenance mechanisms, as we did not instruct participants specifically how they should maintain the memory items (i.e., by rehearsal, refreshing, or elaboration). Future studies can build on these results and investigate in more detail which of the specific maintenance mechanisms interfere with response selection in the interruption task.

Further, while our study focused on cognitive mechanisms under controlled conditions with well-established tasks (i.e., free recall memory task with word lists and spatial Stroop task), future research could build on these findings by replicating them with tasks that are ecologically closer to the complexity of task interruptions in applied settings, such as healthcare (i.e., interrupting more complex information with more urgent tasks). Also, it would be important to investigate which kind of information (i.e., more or less important information for patient safety) would get lost.

Besides these points, for future research it would be important to not only investigate the effect of the interruption position, but also the encoding position of information (i.e., are memory items more or less likely to be recalled if encoded before vs. after the interruption or is the serial position of memory items within the list important?). Moreover, as we showed that the interruption context also impairs interruption task performance itself, future task interruption research should not neglect an analysis of the interruption tasks, to fully understand the potential trade-offs between the primary task and interruption task and the influencing factors for the disruptiveness of interruptions in both tasks.

## Summary and conclusion

In sum, our results showed interruption costs in an auditory-verbal free recall task with a delayed memory test as ongoing primary task and different visual-manual RT tasks as intervening interruption tasks. Specifically, high-workload and late interruptions lead to a significant decrease in recall accuracy compared to low-workload and early interruptions. In addition, performance of the high-workload intervening interruption task itself was negatively impacted by the interruption context relative to performing the task in a single-task condition. We propose that memory maintenance (i.e., rehearsal, refreshing, or elaboration) in the primary memory task interferes with response selection in the interruption task. As the high-workload interruption task involves a stronger decision component and conflict resolution (i.e., incongruent trials) compared to the low-workload interruption task, increased interference is observed with increased interruption task workload. Further, we assume that early interruptions are less harmful than later ones because less primary task information needs to be maintained during interruption task processing.

Overall, our study provides evidence for harmful effects of task interruptions on auditory-verbal LTM performance and shows that the workload in the interruption task and the interruption position modulates the disruptiveness of an interruption. Further, our results highlight the importance of minimizing interruptions or designing strategies to reduce the harmful effects of task interruptions, to avoid communication errors and information loss in critical work environments like healthcare.

## Figures and Tables

**Fig. 5:**
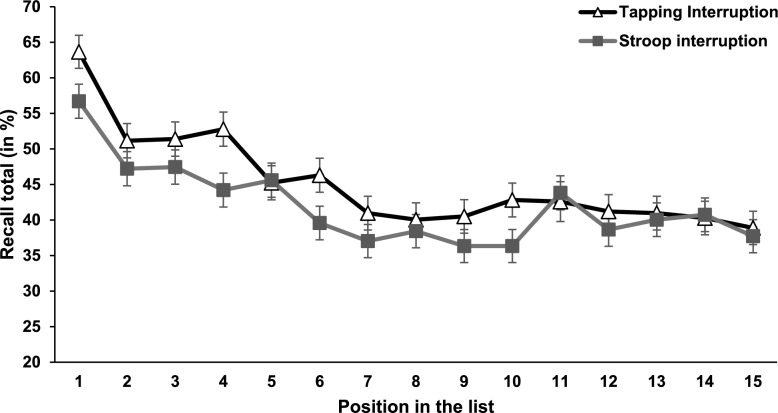
Recall total (in %) as a function of serial position in the word list

## Data Availability

The datasets generated and analyzed during the current study are available in the OSF repository (https://osf.io/s3eby/overview?view_only=4a4d7d7050144eb6acc682260bed14d0).

## Appendix

On an exploratory basis, we analyzed the recall probability of memory items depending on their serial position in the word list in both the tapping task interruption condition as well as spatial Stroop interruption condition, to ensure that differences between the conditions are not due to serial position effects in just one of the conditions. The serial position effect describes that in free recall memory tasks, items that are positioned at the beginning (i.e., primacy effect) or end (i.e., recency effect) of a list are more likely to be recalled than items in the middle of a list (see e.g., Murdock, 1962). Figure 5 shows an overview of recall probabilities of memory items depending on their serial positions in the 15-element word list﻿. 

To analyze serial position effects, we computed three different categories for the memory items: beginning, middle, and end of list items. The recall probability of memory items with positions one to three was used to compute the category beginning of the list, positions seven to nine for the category middle of the list, and positions 13 to 15 to compute the category end of the list. We conducted a 2 × 3 ANOVA with the independent variables interruption task complexity (low-workload tapping task vs. high-workload spatial Stroop task) and serial position (beginning vs. middle vs. end of list) for recall total. Results showed a significant main effect of interruption task complexity (*F*(1,71) = 5.24, *p* =.025, η_p_^2^ =.069), showing reduced recall total of beginning, middle, and end of list memory items when being interrupted by the spatial Stroop task compared to the alternating tapping task (42.4% vs. 45.3% or 6.4 words vs. 6.8 words). Moreover, we also found a significant main effect of serial position (*F*(2,142) = 42.19, *p* <.001, η_p_^2^ =.373). Post hoc pairwise comparisons showed a significant difference between the serial positions beginning (52.9% or 7.9 words) and middle (38.9% or 5.8 words; *p* <.001), as well as beginning (52.9% or 7.9 words) and end (39.8% or 5.8 words; *p* <.001), indicating a primacy effect.

There was no significant difference between the serial positions middle and end (*p* =.588). Crucially, the interaction between interruption task complexity and serial position was not significant (*F*(2,142) = 1.02, *p* =.364).

To summarize, our findings showed higher recall probabilities for beginning of list memory items compared to middle and end of list items, indicating a primacy effect. This primacy effect was not modulated by the different interruption task complexity conditions.

## Abbreviations

ANOVA: Analysis of variance
LTM: Long-term memory
RT: Reaction time
WM: Working memory

## Footnotes

1 Task interruptions can be either unexpected (e.g., Altmann & Trafton, 2007; Brixey et al., 2007) or scheduled and predictable (e.g., at a specific point in time, Gresch et al., 2021; or after a specific action, Powers & Scerbo, 2023; see also McFarlane, 1998, for different dimensions of task interruptions).

2 Our experimental approach might resemble a simplified version of a complex span task, where, e.g., a recall task is combined with a math operation task to examine WM capacity (see e.g., Redick et al., 2013). Yet, while there are some similarities (e.g., a memory task and processing task combined), there are more differences between the approaches (e.g., number of interruptions, interruption duration, interruption timing, type of memory test etc.).

3 Working memory (WM) is a limited system, which holds information immediately accessible for processing, but information decays rapidly (Baddeley, 1986). In contrast, long-term memory (LTM) is understood as an (unlimited) permanent store from which information can be retrieved for later use (Tulving, 1972).

4 The congruency effect occurs due to a processing conflict at target identification and response selection (e.g., the word “left” on the right side of a screen activates both a left and right key press response; Botvinick et al., 2001).

5 On an exploratory basis, we also analyzed if interruption task performance was affected by the interruption position by conducting a 2 × 2 × 2 ANOVA with the independent variables interruption position, congruency, and central code for RT and error rates. We could not find any significant main effect or interaction of interruption position on RT or error rates (all *F*s < 2.38, all *p*-values <.129).

6 Mental representation of cognitive and motor requirements to achieve a specific task goal (see, e.g., Kiesel et al., 2010; Koch et al., 2018).

7 In Hensen et al. (2024), we also compared whether both the spatial and verbal stimulus material groups were comparable in their general cognitive abilities by testing WM capacity in both groups. We could not find significant group differences, but overall performance was worse with the verbal stimulus material compared to the spatial stimulus material.

## Declarations

### Open practice statement

The datasets generated and analyzed during the current study are available in the OSF repository (https://osf.io/s3eby/overview?view_only=4a4d7d7050144eb6acc682260bed14d0). There was no pre-registration.
